# Solvent Transfer—Efficiency of Risk Management Measures

**DOI:** 10.1093/annweh/wxx090

**Published:** 2017-11-19

**Authors:** Katharina Bluemlein, Manfred Elend, Tim Meijster, Alison Margary, Rosalie Tibaldi, Stefan Hahn, Susanne Hesse

**Affiliations:** 1 Fraunhofer Institute for Toxicology and Experimental Medicine, Germany; 2 Shell Health, Shell International B.V., Risk Science Team, The Netherlands; 3 Shell Health, Shell International Ltd, Risk Science Team, UK; 4 ExxonMobil Biomedical Sciences, Inc., USA

**Keywords:** containment, drum pump, emission reduction, extract ventilation, REACH, risk management measures, solvent transfer

## Abstract

A series of laboratory simulations were conducted in order to determine the airborne protection that might be afforded by different combinations of workplace exposure controls typically encountered when handling volatile solvents (e.g. solvent transfer). These conditions, referred to as risk management measures (RMMs) under the Registration, Evaluation and Authorisation of Chemicals Regulation (REACH), are typically described using standard phrases in safety data sheets [and specifically those of the European Phrase Catalogue (EUPhraC)]. Ethanol was used as a model compound and its emissions were monitored continuously with a portable IR spectrometer at 3000 cm^−1^. The average emission reduction performance of the investigated RMMs (e.g. containment, extract ventilation, drum pump) exceeded 90%. They present suitable ways to reduce airborne solvent exposure in a workplace and confirmed the initial expectations derived at by the European Solvents Industry Group (ESIG) and the European Centre For Ecotoxicology and toxicology of Chemicals (ECETOC) Targeted Risk Assessment (TRA) model.

## Introduction

Under the European Registration, Evaluation, Authorisation and Restriction of Chemicals Regulation (REACH), chemical safety assessments (CSAs) need to be developed for registered hazardous substances. These CSA’s include an assessment for workers, consumers and the environment, of the exposures arising from all the various use of the substance. The exposure assessment considers those exposure controls (risk management measures, RMMs) that need to be in place to manage exposures to acceptable levels (and specifically to less than the relevant Derived No Effect Level (DNEL) and/or Predicted No Effect Concentration (PNEC) for the substance). Although ECHA Guidance R.14 (paragraph: 6.2) indicates a preference for the use of measured exposure data, because of the general paucity of such data across all uses of substances, registrants are encouraged to use suitable workplace exposure models (ECHA Guidance, 2016).

Solvents are ubiquitous chemical substances found in almost all sectors of everyday life. Solvent exposure can occur via the oral, dermal, and inhalation route, with inhalation often posing the most relevant exposure route due to the volatile nature of most (organic) solvents. Handling of solvents that lead to exposure of humans at levels that may pose a health risk invariably requires the implementation of RMMs to reduce solvent exposure. In some occupational environments where large amounts of solvents are handled, e.g. during solvent transfer, the implementation of RMMs is essential to reduce solvent emissions into the work environment.

Established occupational hygiene practices seek to apply a hierarchy of preferred measures to control exposures to solvents (ESIG, 2016) and this is also reflected in ECHA Guidance R.14 (paragraph 5.2; ECHA Guidance, 2016).

Where RMMs need to be implemented (either alone or in combination), REACH requires such RMMs to be communicated as part of an Annex to the safety data sheet (SDS). This, in turn, demands that chemical suppliers have some understanding on the likely effectiveness of the types of RMM that may be used to manage exposures to their products, as well as providing valuable information for the users of such materials. In support of the introduction of REACH, ESIG identified a number of RMMs that are typically deployed to control emissions of solvents and described these in the form of standard sentences in order that they could be consistently communicated, when appropriate, in SDSs (in the various EU languages). For some of these RMMs (CONCAWE, 2012), using available literature and the experiences of solvent suppliers, a level of effectiveness was identified commensurate with what might reasonably be anticipated in a workplace. This included the proper installation and maintenance of the RMMs, but also recognized that the assumed effectiveness will be lowered by improper installation and/or use. However, supporting data for the effectiveness of these RMMs were lacking. In particular, ESIG identified that the use of drum pumps for filling procedures, various levels of containment in combination with ventilation (ACGIH, 2013) and draining and flushing procedures before cleaning and maintenance operations (van Wagenen, 1981; CONCAWE, 2012) were all forms of RMMs whose effectiveness required better characterization. As a result, a project was initiated to review currently available information on the effectiveness of such RMMs and undertake a range of experiments to generate data on the emission reduction of these, and similar, RMMs. Laboratory-based simulations scenarios, reflecting real life use as closely as possible, were set-up for solvent transfer processes using ethanol as model-compound. Here, all steps from the approach via the conduction of the simulations to the final evaluation of the results regarding the efficiency of the implemented RMMs are described.

## Methods

### Literature research

In the course of the literature research, the single datasets within the ECEL database (Exposure Control Efficacy Library; Fransman et al., 2008) have been re-evaluated considering solvent exposure and RMMs of interest. The results have been supplemented by more recently published scientific literature gathered via reputable search engines (WebOfScience, SciFinder, Scopus; see *Literature Research—Additional Information* in the Online Supplementary Material, available at the *Annals of Work Exposures and Health*).

In addition, drum pump manufacturers and a number of representatives from relevant industry areas (e.g. formulators, metalworking fluid sector) were contacted and asked for general information on solvent handling and RMMs as well as quantitative exposure data.

### Experimental

In this study, standardized laboratory-based experiments were developed to simulate several use scenarios typical for solvents. Various typical RMMs were included in the experiments to assess their effectiveness, both in isolation and in combination with other measures. Based on the EUPhraC phrases (eSDScom alliance), nine solvent-related exposure scenarios were identified, investigated in laboratory simulations, and the airborne solvent emission compared to respective baseline scenarios (#1 and #8, Table 1). Baseline scenarios, were considered as worst-case situations, with no RMMs in place. The details of the nine scenarios and their translation into experimental simulations are provided in Table 1.

**Table 1.** EUPhraC Phrases and their implementations in laboratory-based simulations (exposure scenarios [ES]).

ES #	EUPhraC Phrase	Scenario/simulation set-up
1		Baseline–gravity transfer (splash loading) from an open container into another open container with no exhaust and ventilation system in place. Outside of fume cupboard.
Gravity transfer		
2	Phrase: E60 ‘Minimise exposure by partial enclosure of the operation or equipment and provide extract ventilation at openings’ *Or* E83: Handle in a fume cupboard or under extract ventilation *Or* E66: Ensure material transfers are under containment or extract ventilation	Open gravity transfer (splash loading) with partial enclosure (inside open walk-in fume cupboard) into a container. Room ventilation and fume cupboard switched on.
3	Phrase: E61 Minimise exposure by extracted full enclosure for the operation or equipment	Open gravity transfer (splash loading) with full enclosure (inside closed walk-in fume cupboard) into a container. Room ventilation and fume cupboard switched on.
4	Phrase: E54 ‘Provide extract ventilation to points where emissions occur’	Gravity transfer (splash loading) from an open container into another open container—application of a local exhaust system (LEV, elephant trunk) and no enclosure (outside fume cupboard). Room ventilation and fume cupboard^a^ switched on.
	Phrase: E66 ‘Ensure material transfers are under containment or extract ventilation’	
Accurate drum pump transfer (submerged loading)		
5	Phrase: E53 ‘ Use of drum pump’^b^ (Phrase: E68, ‘Restrict area of openings to equipment’)^c^	Drum pump transfer (lids on containers) with no exhaust and no room ventilation—accurate use of drum pump (submerged loading). Outside of fume cupboard.
6	Phrase: E66 ‘Ensure material transfers are under containment or extract ventilation.’	Drum pump transfer (lids on containers) with partial enclosure (inside open walk-in fume cupboard)—accurate use of drum pump (submerged loading). Room ventilation and fume cupboard switched on.
	E60: Minimise exposure by partial enclosure of the operation or equipment and provide extract ventilation at openings (Phrase: E68, ‘Restrict area of openings to equipment’)^c^	
	Phrase: E53 ‘Use of drum pump’^b^	
7	Phrase: E54 ‘Provide extract ventilation to points where emissions occur’	Drum pump transfer (lids on containers), room ventilation and a local exhaust ventilation system in place (elephant trunk)^a^—accurate use of drum pump (submerged loading). Outside of fume cupboard.
	Phrase: E66 ‘Ensure material transfers are under containment or extract ventilation.’ (Phrase: E68, ‘Restrict area of openings to equipment’)^c^	
	Phrase: E53 ‘Use of drum pump’^b^	
Drain and flush		
8	E65: Drain down system prior to equipment break-in or maintenance *Or* E81: Drain or remove substance from equipment prior to break-in or maintenance	Base configuration for scenario 9: drained container without flushing with no exhaust and ventilation system in place. Outside of fume cupboard.
9	Phrase: E55 ‘Drain down and flush system prior to equipment break-in or maintenance.’	Flushed container with no exhaust and no room ventilation system in place. Outside of fume cupboard.

The baseline scenarios describe the worst-case situation for solvent transfer (ES 1) and drain and flush activities (ES 8).

^a^The operating fume cupboard was an integral part of the LEV.

^b^Here, the experimenter assumed submerged loading as good practice.

^c^Standard handling for solvents.

#### Equipment and chemicals

**Figure 1.**
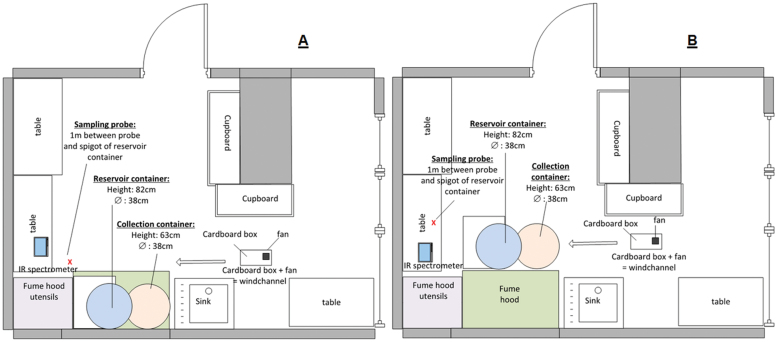
Schematics of experimental set-ups—A: simulations conducted inside of the fume cupboard; B: simulations conducted outside of the fume cupboard.

Throughout all simulations, bioethanol (Kaminethanol; PN: 10295; 96.6 % ethanol, vapour pressure: 5900 Pa) was used. The general experimental set-up of the conducted simulations is given in Fig. 1. A list of all the applied equipments can be found in the Supplementary Table S2 in the Online Supplementary Material (available at the *Annals of Work Exposures and Health*).

#### Experimental set-up and data acquisition

The airborne ethanol concentration released during the activities was continuously monitored using a portable IR-spectrometer (Asynco). Its concentration at the IR sampling probe was recorded every 20 seconds at a wave number of 3000 cm^−1^.

The standardized conditions of the simulated exposure scenarios comprised the IR probe always being positioned at approximately the same distance from the exposure source (100 cm) and always at the same height from the floor (95 cm). A static sampling approach was preferred over sampling of the breathing zone of an individual worker in order to keep the variations between simulations as low as possible.

Additionally a fan was installed, constantly mixing the air in the room and thereby keeping the impact of uncontrolled air movement (e.g. the movement of personnel involved with the experiment) in the room to a minimum. This resulted in an improved reproducibility of the experiments. For each scenario, replicate simulations were conducted with a minimum of three repetitions. Care was taken before starting a new simulation to confirm the ethanol background level in the room had been reached.

The sampling set-up was chosen such that ethanol vapours released during each solvent transfer process were directed towards the IR probe by a fan, keeping the inter-experimental variation of the respective simulation scenarios as low as possible. For all experiments conducted inside the fume hood, the IR probe was positioned outside of the fume hood as only the airborne ethanol outside of the hood is relevant for workplace exposure scenarios. The positioning of the solvent transfer equipment did have an influence on the absolute readings. In terms of data evaluation, this effect was overcome by considering the extrapolated rather than the absolute values (please see data evaluation).

All simulations were conducted in a room (45 m^3^) holding a 2-451-GAND walk-in fume hood with vertical sashes. The efficient operation of this fume hood required an additional air supply into the room to prevent the build-up of negative pressure in the room (an air-exchange rate of ~14–18 per hour in the room). This air supply was provided by the fixed-room ventilation system which was switched on during the simulations requiring the operation of the fume hood. The room ventilation circulated the additional air via an inlet (~1000 m^3^ h^−1^) and an outlet (~600 m^3^ h^−1^). In the event of the fume hood being switched on the outlet valve of the room, ventilation was closed and all air supplied by the room ventilation was removed via the fume hood (~1100–1200 m^3^ h^−1^). In the simulation scenarios, where the efficiency of local exhaust ventilation (LEV) systems was addressed, a self-assembled LEV was created using the fume hood ventilation system as the source for the extracted air, providing a face velocity of ~1 m s^−1^. In these cases, the vertical sash of the fume hood was closed and sealed at the bottom, only leaving a small opening to provide the necessary air inflow for the operation of the LEV. The other opening was the LEV capture hood itself (Fig. 3). The face velocity of ~1 m s^−1^ obtained this way was in the upper range of the recommended face velocity range of 0.5 to 1.0 m s^−1^ for liquid transfer processes (HSE 2011; HSE Control Guidance Sheet 212).

In all solvent transfer simulations, 50 l of ethanol were transferred either by: (i) gravity (splash loading) scenarios #1 to #4 with a product flow rate of ~12.5 l min^−1^, or (ii) correct use of a drum pump (submerged loading) scenarios #5 to # 7 with a product flow rate of ~50 l min^−1^. The scenarios addressing draining and flushing were simulated by: (i) Rinsing the inside surface of the collection container with 1 × 5 l ethanol in a separate room and removing the lid of the drum in the laboratory directly afterwards, representing a just drained container (scenario #8) and (ii) Rinsing the inside surface of the collection container first with 1 × 5 l ethanol followed by rinsing them with 2 × 10 l water in a separate room and removing the lid of the drum in the laboratory directly afterwards, representing a flushed container (scenario #9).

#### Data evaluation

**Figure 2.**
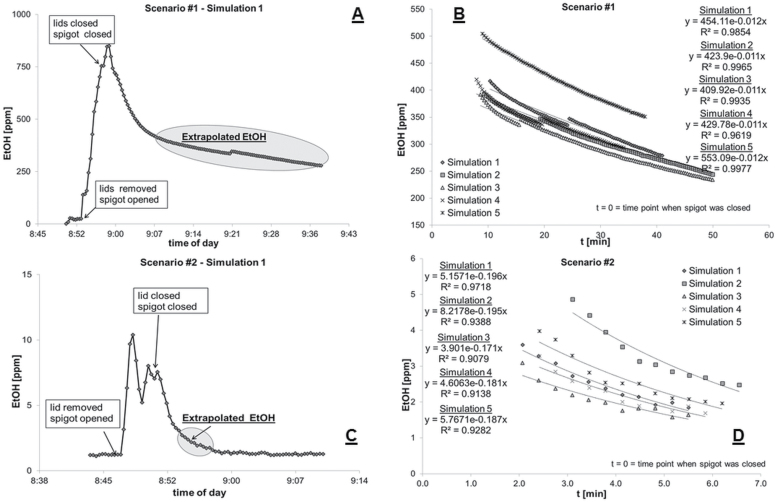
Examples of recorded course of airborne ethanol concentration for exposure scenario #1 (A) and #2 (C) as well as the respective graphs for the calculation of EEC (B: exposure scenario #1 and D: exposure scenario #2).

In terms of data evaluation, a simulation was defined as the time window in which the solvent transfer took place and the resulting measured ethanol vapour concentration at the sampling probe had fallen to the respective background concentration (Fig. 2C) or was observed to have stabilized (Fig. 2A). The overall time for a simulation varied with the scenarios, depending on the type of ventilation, solvent transfer etc. (Fig. 2).

The ethanol concentration at the sampling probe was influenced by various factors such as re-positioning of equipment between simulations, unavoidable movements of the experimenters in the room etc. The unpredictability of these factors made the readily available peak ethanol concentration an unsuitable parameter for assessing the effectiveness of the different RMMs. The same applies for the average ethanol concentration during the process of the solvent transfer (between opening and closing of the spigot) or any other values based on the direct readings at the IR probe. To overcome these factors, the extrapolated ethanol concentration (EEC) was used for assessing the level of ethanol vapour resulting from the simulation. The EEC is regarded as the value that comes closest to the hypothetical mean ethanol concentration in the room, built up during the respective solvent transfer processes. The better the RMM efficiency, the lower the EEC. Therefore, the EEC is a good indirect measure for the efficiency of the applied exposure control measure. On the positive side, the EEC averages out the effect of uncontrolled movement and vapour pockets causing short peaks in the exposure concentration measured over time, providing data with fairly low coefficients of variation (C.V.). The C.V. for EEC varied between 7.7 and 30%, whereas data based on the direct reading at the sampling probe (average ethanol concentration during the process of the solvent transfer) had C.V. up to 87% (data not shown).

The determination of the EEC was achieved by plotting selected data points from the descending part of the ethanol exposure graphs against time (in minutes); where time point zero corresponds to the time point when the ethanol transfer was completed. The resulting graph reflects an exponential decay which can be fitted by a trendline with the overall formula: *y* = *y*_0_ exp(−k·*t*) with *y*_0_ being the EEC. The correct choice of the selected data points for this trendline is crucial and is determined by the constant k of the respective graph. Multiplying k with 60 min results in the air-exchange rate (per hour) in the room. To allow a comparison of the data this parameter had to be kept constant for the given circumstances—room ventilation off (air-exchange rate ~1) or on (air-exchange rate 11 ± 1; Fig. 2).

#### Calculation of effectiveness of a given RMM

For each exposure scenario, the minimum, the maximum, and the mean observed emission reduction were calculated according to the following equations:

1. Minimum observed emission reduction in %: (1 − (E_H,x_/E_L,b_)) * 100
2. Maximum observed emission reduction in %:(1 − (E_L,x_/E_H,b_)) * 100
3. Mean observed emission reduction in %: (1 − (E_M,x_/E_M,b_)) * 100.

E_L,b_: lowest emission value (=EEC) for baseline scenario; E_H,b_: highest emission value for baseline scenario; E_M,b_: mean arithmetic average emission value for baseline scenario; E_H,x_: highest emission value for scenario #X; E_L,x_: lowest emission value for scenario #X; E_M,x_: mean arithmetic average emission value for scenario #X.

## Results

The literature research conducted at the beginning of the study identified a number of literature sources containing exposure data but their value was limited. The ECEL database for example despite it comprising many datasets did not contain any representative information for solvents. Information gathered via interviews with drum pump manufacturers and representatives from relevant solvent industry areas were only of qualitative value. Overall, publicly available information was found to be insufficient to evaluate the effectiveness of common solvent-related RMMs (ESIG, 2016).

Thus, it was decided to further evaluate the influence of RMMs on inhalation exposure via experimental studies.

Addressing the relative effectiveness allowed the implementation of standardized conditions (i.e. stationary sampling, establishment of an artificial wind channel) rather than having to simulate workplace monitoring procedures. The latter would direct its attention to the airborne ethanol concentration in the breathing zone of a worker, resulting in highly variable results.

Gravity transfer with no RMMs implemented was established as the baseline scenario (#1) to which all gravity transfer scenarios (#2 to #4) and all drum pump transfer scenarios (#5 to #7) were compared in order to assess their effectiveness (Table 1). For the drain/flush scenario (scenario #9), a separate baseline scenario was developed (scenario #8).

### Gravity transfer

The baseline (worst case) scenario (#1) involving the gravity transfer of solvents into an open drum measured the highest airborne ethanol concentration with an average concentration of 454 ppm (Table 2).

**Table 2.** Effectiveness of selected RMMs for gravity (#2 to #4) and drum pump (#5 to #7) transfer in comparison to baseline scenario (#1).

Scenario #		1		2		3		4		5		6		7
		Baseline		Gravity transfer						Drum pump transfer				
Description		Baseline–gravity transfer		Vented open gravity transfer with partial enclosure		Vented open gravity transfer with full enclosure		Gravity transfer— local exhaust system and no enclosure		Drum pump transfer —no exhaust and ventilation system in place—submerged loading		Vented drum pump transfer—submerged loading		Drum pump with full enclosure and a local exhaust system in place—submerged loading
Simulation		Extrapolated EtOH [ppm]												
1		454		5		NoE		12		33		3		6
2		424		8		NoE		14		27		2		5
3		410		4		NoE		13		28		2		5
4		430		5		NoE		NA		NA		NA		NA
5		553		6		NoE		NA		NA		NA		NA
MV		454		6		NoE		13		29		2		5
Emission reduction														
Minimal [%]		NA		98.0		NA		96.5		91.9		99.3		98.6
Optimal [%]		NA		99.3		NA		97.9		95.2		99.7		99.1
Mean [%]		NA		98.8		NA		97.1		93.5		99.5		98.9
Efficiency originally suggested by ESIG and TRA [%]		NA		Prof: 80		Prof: 90		Standard ECETOC TRA efficiency:		All uses: 80		All uses: 80		Standard ECETOC TRA efficiency:
				Industrial: 90		Industrial: 95		General ventilation: 30/70 (good/enhanced); LEV: 75–95 (general); 80–95 (PROCs 8a/8b/9)						General ventilation: 30/70 (good/enhanced); LEV: 75–95 (general); 80–95 (PROCs 8a/8b/9)

NA = not applicable; NoE = no observable increase in ethanol concentration in the room.

The effectiveness of a vented partial enclosure was studied in scenario #2 (Table 2; see also Supplementary Table S3 in the Online Supplementary Material, available at the *Annals of Work Exposures and Health*). This scenario was simulated by including the gravity transfer activity inside a vented walk-in fume hood with the vertical sash remaining fully open. The average extrapolated ethanol vapour concentration was 6 ppm, equating to in a mean reduction in air concentration of 98.8 %.

Scenario #3 was also carried out inside the fume hood but with the vertical sash closed, resembling an enclosed ventilated gravity transfer of ethanol. In this scenario, the ethanol vapour emission was such that no significant rise in the ethanol concentration over the course of the individual simulations was observed compared to the background concentration (Table 2; see also Supplementary Table S3 in the Online Supplementary Material, available at the *Annals of Work Exposures and Health*). Therefore, the effectiveness is assumed to be greater than 99%.

**Figure 3.**
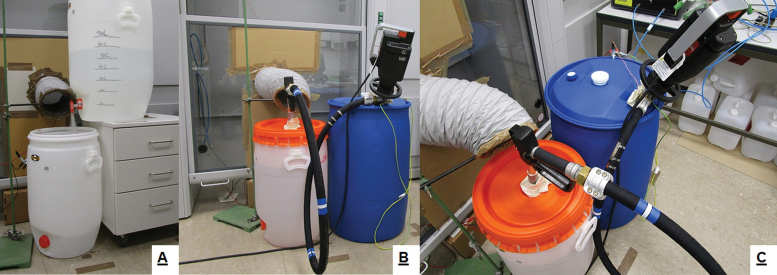
Self-assembled local exhaust ventilation system (LEV, ‘Elephant trunk’) using a fume hood. A: Gravity transfer; B and C: drum pump transfer.

For scenario 4, an LEV system (‘Elephant Trunk’) with a face velocity of ~1 m s^−1^ was built by utilizing the fume hood (Fig. 3). The LEV was positioned at the vapour emission point (i.e. the spigot of solvent reservoir container) and resulted in a calculated effectiveness of 96.5–97.9%. The extent that the general ventilation in the room itself contributed to the observed effectiveness is unknown. Additional experiments are necessary to shed light on the possibility of a combined effectiveness.

### Drum pump transfer

Scenario #5 involved the use of a drum pump to transfer the solvent. In this scenario, the only control for the vapour emission during the solvent transfer was the drum pump itself (correctly used to minimize the generation of vapour by avoiding the splashing of the solvent during the transfer, known as ‘submerged’ loading). There was no other control measure applied such as the fume hood or LEV, and also the general room ventilation was disabled. The average EEC for scenario #5 was 29 ppm, which equates to an emission reduction of 91.9 to 95.2% when compared to the baseline scenario #1 (Table 2; see also Supplementary Table S3 in the Online Supplementary Material, available at the *Annals of Work Exposures and Health*).

Scenario #6 involved a drum pump transfer within a vented partial enclosure. This scenario was simulated by moving the solvent transfer activity into a vented fume hood with the sash remaining fully open and resulted in an average EEC of 2 ppm, which equates to an emission reduction of 99.3 to 99.7% (Table 2; see also Supplementary Table S3 in the Online Supplementary Material, available at the *Annals of Work Exposures and Health*). The overall effectiveness of the RMMs applied in scenario #6 is similar to the emission reduction seen in scenario #3 (vented open gravity transfer with full enclosure). Comparing scenario #6 to the basic drum pump scenario (#5) the incorporation of an exhaust system within a partially enclosed cabinet yielded in an additional emission reduction of 89.6–93.2% related to partial enclosure.

In scenario #7, the effect of using LEV (‘elephant trunk’) was investigated combined with the drum pump transfer, as it was done for the gravity transfer scenario (#4; Fig. 3). The comparison of the scenarios #5 and 7 to the baseline scenario #1 showed that the emission reduction effectiveness of the drum pump (93.5 %) could be further increased. The application of the assembled LEV (LEV could only be operated in conjunction with the fume hood) in combination with room ventilation (Table 2; see also Supplementary Table S3 in the Online Supplementary Material, available at the *Annals of Work Exposures and Health*) showed and effectiveness in reducing the solvent emission of 98.9%.

### Drain and flush application

The effectiveness of flushing a container on solvent emission reduction could not be assessed by referring to scenario #1 as the baseline scenario. The baseline of a just drained container (scenario #8) was established to serve as a suitable baseline scenario. A drum was rinsed with ethanol ensuring the surfaces inside were fully coated and the drum closed. This was done in a separate room to the laboratory where the measurements were being carried out. The closed drum was then moved into the laboratory and the lid removed from the drum with the subsequent airborne ethanol level recorded. A mean EEC of 53 ppm was determined for this baseline scenario (Table 3).

**Table 3.** Effectiveness of flushing container on emission reduction.

Scenario #		8		9
Description		Base configuration for scenario 9—drained container without flushing with no exhaust and ventilation system in place		Flushed container with no exhaust and ventilation system in place
Simulation		Extrapolated EtOH [ppm]		Mean^a^ EtOH [ppm]
1		59		2.5
2		54		3.1
3		46		2
MV		53		2.5
		Emission reduction		
Minimum [%]		NA		93.2
Maximum [%]		NA		96.6
Mean [%]		NA		95.2
Efficiency originally suggested by ESIG and TRA [%]		NA		Industrial: 90

^a^Average of all concentration data points between opening and closing lid.

The flushed container simulation involved rinsing the inside surfaces of the drum with 5 l of ethanol before rinsing it twice with 10 l of water (scenario #9). Again, this was done in a separate room and the closed drum was then moved back into the laboratory, the lid removed and the airborne ethanol concentration recorded. The measurement of the increase in the airborne ethanol concentration emitted from the flushed container simulations did not allow the determination of an EEC. Hence, the exposure concentration was calculated as the average concentration of all recorded data points between opening and closing the lid of the flushed container. The effectiveness of reducing the solvent emission by rinsing a just drained container was determined as 93.2 to 96.6% (Table 3; see also Supplementary Table S3 in the Online Supplementary Material, available at the *Annals of Work Exposures and Health*).

## Discussion

While it is preferable to make assessments of the effectiveness of RMMs in situ in the workplace, this is not straightforward and without significant resources, it is difficult to make comparisons between different RMMs at different workplaces. For this reason, the RMMs were evaluated under simulated workplace conditions. Care was taken that these conditions represent what might reasonably be encountered in a workplace, rather than some idealized situation. However, as the focus of this study was the assessment of RMM efficiencies, the primary interest was the reduction in airborne solvent emission concentration achieved by implementation of (combinations of) RMM compared to a baseline scenario. The absolute emission concentrations were of secondary importance. As the scope of this study was the assessment of RMM efficiencies during the solvent transfer process, the data presented here do not consider any other activities such as equipment change over that might also pose a source of solvent exposure. For example, in the case of the drum pump scenarios only the filling itself was evaluated. While exposure during the actual use of a drum pump is very low, peaks of exposure may occur during removal of the pump or change to the next drum. In addition to inter-scenario comparison within the study results, also a general non-statistical comparison was made to the underlying assumption in the ECETOC TRA tool and suggestions previously made by ESIG.

Splash loading is a method of gravity transfer which can be expected to generate high airborne solvent concentrations in the air. Additionally, the collection container may have a relatively large opening contributing to potentially greater airborne solvent concentration during such a transfer. These characteristics of a gravity transfer, lends it to being a reasonable worst-case scenario for generating airborne solvent emissions when no RMMs are implemented, and a suitable baseline against which to compare the effectiveness of various controls.

As explained, the effectiveness of various RMM (combinations) were evaluated against the above described baseline scenario. The implementation of the RMMs described by EUPhraC Phrases E60, E61, E66, and E83 (see Table 1) addressed the enclosure of the operation and/or equipment (scenarios #2 and #3), in the laboratory-based simulations. Results showed average emission reductions of above 98% (Table 2, see also Supplementary Table S3 in the Online Supplementary Material, available at the *Annals of Work Exposures and Health*). These were in good agreement with the predictions made by ESIG and TRA, which suggested 80 (professional) to 90 (industrial) % efficiency for the application of E60, and 90 (professional) to 95 (industrial) % emission reduction when applying an extracted full enclosure for the operation and equipment (E61). However, full containment of the process of solvent transfer within, for example, a vented fume hood may not always be an option in practice. A more conventional method to reduce emissions at source is via a local exhaust ventilation system (EUPhraC Phrases E54 and E66—Table 1) represented by scenario #4 (Table 2; see also Supplementary Table S3 in the Online Supplementary Material, available at the *Annals of Work Exposures and Health*). The average efficiency of the self-assembled LEV was determined as 97.1%, which was also in good agreement with 75 to 95% predicted by ESIG and TRA. The simulation might have been affected by the general ventilation of the room which was necessary for the faultless functioning of the LEV. General room ventilation itself is regarded as an RMM with an expected efficiency of 30 to 70% in ECETOC TRA v.3. Even though its influence on the measured LEV efficiency is considered to be negligible in this experimental study, it cannot be ruled out and further experiments to address this issue are advised. The data obtained show that placing the process in an enclosed and vented environment results in a marginally higher emission reduction than applying LEV alone, suggesting an interchangeable use of these RMMs (98.8% compared with 97.1%).

The application of a drum pump was an effective RMM as it reduced the transfer time by a factor of ~4, compared to the gravity transfer scenarios. In addition to this, the drum pump transfer allowed submerged loading via a lance in contrast to splash loading during gravity transfer. These two points are considered to be the major factors in contributing to the emission reduction during solvent transfer. A further factor influencing the effectiveness of the control is the size of the drum openings. For the gravity transfer, the lids of the source and receiving drums had been removed so the transfer was ‘open’, whilst for the drum pump transfer they were significantly smaller and may be considered an additional RMM. As for the gravity transfer, the experimentally determined RMM efficiencies for the drum pump-related EUPhraC phrases (E53, E60, E66, E68; scenarios # 5 and 6, Table 2, see also Supplementary Table S3 in the Online Supplementary Material, available at the *Annals of Work Exposures and Health*) were with values above 91.9% in good agreement with the 80% predicted by ESIG and TRA. The simulation regarding the combination of drum pump transfer and LEV (E53, E54; E66, E68, scenario # 7, Table 2; see also Supplementary Table S3 in the Online Supplementary Material, available at the *Annals of Work Exposures and Health*) showed an averaged emission reduction of above 98.1% which is higher and in good agreement with the predicted 75–85%. Again, as for the respective LEV-gravity transfer simulation, the impact of the necessary general room ventilation was unknown. The use of a drum pump without having the process under containment or any ventilation in place resulted in an emission reduction of above 91.9%, which in some instances might be sufficient to make it a suitable alternative to LEV.

In this study, all simulations involving a drum pump applied submerged loading. This approach might not always be implemented in situ at workplaces, although unlikely for the transfer of solvents for which the prevention of static charge build-up is a necessary control for flammability and standard operating practice. The use of a drum pump can result in a high pressure solvent transfer, depending on the specifications of the pump. High pressure solvent transfer has the potential for an increased aerosol fraction in the exposure atmosphere due to the higher impact velocity of the transferred solvent onto the solvent surface in the receiving container: the more droplets/aerosol the higher the solvent surface area and the higher the solvent surface area the more solvent can evaporate. If such a transfer is not contained, the solvent exposure might be as high as during gravity transfer or even higher. In such an instance, a drum pump is merely a tool to speed up the solvent transfer (increase in work efficiency) but cannot be considered as a RMM; unless the exposure duration is considerably reduced compared to gravity transfer, reducing the overall exposure. The change from gravity transfer to drum pump transfer increased the product flow from 12.5 l min^−1^ up to 50 l min^−1^, both assignable to the 10–100 l min^−1^ product flow category in the Advanced REACH Tool (ART 1.5; Fransman et al. 2011). A direct comparison between the two product flow rates regarding solvent emission could not be made as other parameters were changed simultaneously. However, the train of thought applied above is also applicable here as an increased solvent transfer rate is achieved by increased transfer pressure, most likely resulting in an increased aerosol fraction. Fransman et al. (2011) also discuss that liquids might interact with surrounding air during the transfer, resulting in vapour release, which increases with the product flow. It is recommended to increase the level of containment with increasing product flow rate and applying submerged loading when and wherever possible.

The experiments regarding the efficiency of draining and flushing of a system prior to break-in or maintenance (E55) which showed an average of 95.2%, confirmed the expected efficiency of 90% (scenario #9, Table 3; see also Supplementary Table S3 in the Online Supplementary Material, available at the *Annals of Work Exposures and Health*).

The experimentally determined RMM efficiencies were in good agreement with the ECETOC TRA estimates, even though mostly higher. Fransman et al. (2008) has already shown that experimental simulations lead to seemingly better efficiencies as field data. This is most likely attributed to the fact that experimental simulations are standardized tests focussing on the technical capabilities of the RMMs rather than taking into account personal variations of people conducting the tasks. The work of Fransman et al. (2008) also brought to light that negative efficiencies can be the result of erroneous implementation of RMMs. In the particular cases of general ventilation and natural ventilation, this is conceivable when the worker is positioned down stream of the emission source, rendering the RMM useless or even dangerous (Fransman et al. 2008). In the light of this, conservative exposure estimates as provided by ECETOC TRA v.3 are desirable in the process of product/substance registration under REACH.

ART 1.5 is a Tier 2 tool providing more advanced exposure estimate values by considering a number of activity-specific factors besides substance-specific parameters (Fransman et al. 2011). In ART, the applied efficiency values for LEV vary, depending on the type, between 50 and 99%, with canopy hoods and unspecified LEV having the lowest assigned efficiency. Excluding the latter two, the ART estimates on LEV efficiency range between 80 and 99%. These predictions are similar to the ones provided by the ECETOC TRA tool (75–95%), which were confirmed by the experimental values obtained in this study (bearing in mind that standardized settings yield better RMM efficiencies than other approaches).

Besides ECETOC TRA and ART other tools, providing occupational exposure estimates, such as Stoffenmanager^®^, MEASE, and EMKG-EXPO are available.

This study did not differentiate the type of setting (professional/industrial). It is expected the transfer activities experimentally evaluated in this study would be similar regardless of whether performed at an industrial site or other professional setting. It may be useful to verify this and examine other transfer tasks carried out by industrial versus professional workers. Another factor that is most likely to affect the effectiveness of RMMs is the level and frequency of training the operator has received whether in an industrial or professional setting.

## Conclusion

The extent to which RMMs affect measured substance vapour concentrations was examined in this study. Based on our review of the literature more could be done to substantiate the effectiveness of emission controls by field studies or designated laboratory-based simulations.

Here, initial simulations enabled investigators to gather necessary empirical data to assess airborne solvent emissions with and without RMMs. Mean emission reductions were generally high, above 90% for most tested (combinations of) RMMs and in good agreement (same order of magnitude) with the values previously suggested by ESIG and the assumptions contained in the ECETOC TRA model. Given the universe of substances, processes and tasks there is much to be done to improve knowledge of exposure conditions and risk management options. This can be achieved in a first step by simulations but should be backed-up by workplace monitoring campaigns, aiming to provide representative data sets for the assessment of various (combinations of) RMMs in different occupational environments where solvents are handled. Hence, the results presented here should be considered merely as indicators for the efficiency of the RMMs investigated in regards to their potential to minimize airborne solvent emission during solvent transfer processes. These values are not necessarily valid for all solvent transfer processes.

## Supplementary Material

Supplementary data are available at the *Annals of Work Exposures and Health* online.

## Funding

This project was funded by Cefic.

## Declaration

The authors declare no conflict of interest relating to the material presented in this article. Its contents, including any opinions and/or conclusions expressed, are solely those of the authors.

## Supplementary Material

wxx090_suppl_Supplementary_Material

## References

[CIT0001] ACGIH. (2013)Industrial ventilation: a manual of recommended practice for design. Cincinnati: ACGIH 28th edn. ISBN: 978-1-607260-57-8/ 2013

[CIT0002] CONCAWE. (2012)Developing human health exposure scenarios for petroleum substances under REACH Report no. 11/12. Available at Developing human health exposure scenarios for petroleum substances under REACH .Accessed 22 August 2017.

[CIT0003] ECHA Guidance. (2016). Version 3.0: August 2016. Guidance on information requirements and chemical safety assessment. Chapter 14. Available at https://echa.europa.eu/documents/10162/13632/information_requirements_r14_en.pdf. Accessed 10 January 2017.

[CIT0004] ESIG. (2016)Verifying the effectiveness of Solvent RMMs Available at http://www.esig.org/layout/uploads/2016/10/2015-12-15_ESIG_RMM_Final_report.pdf. Accessed 10 January 2017.

[CIT0005] eSDScom alliance (2013) Available at https://www.esdscom.eu/english/euphrac-phrases/download/. Accessed 10 January 2017.

[CIT0006] Fransman W, Schinkel J, Meijster T(2008)Development and evaluation of an exposure control efficacy library (ECEL). Ann Occup Hyg; 52: 567–75. 18703542 10.1093/annhyg/men054

[CIT0007] Fransman W, Van Tongeren M, Cherrie JW(2011)Advanced Reach Tool (ART): development of the mechanistic model. Ann Occup Hyg; 55: 957–79. 22003239 10.1093/annhyg/mer083

[CIT0008] HSE. (2011). Controlling Airborne Contaminants At Work A Guide to Local Exhaust Ventilation (LEV). 2^nd^ edn. ISBN: 978 0 7176 6415 3

[CIT0009] HSE. (2013)Control Guidance Sheet 212. Drum Filling.

[CIT0010] Van Wagenen H (1981)Control of emissions from seals and fittings in chemical process industries. NIOSH; 1–61:81–118.

