# Supplementary material for: Solvent Transfer—Efficiency of Risk Management Measures
Source: Ann Work Expo Health. 2017 Nov 19;62(1):112–23. doi: 10.1093/annweh/wxx090 (PMC6788583; doi:10.1093/annweh/wxx090)
Supplement: wxx090_suppl_Supplementary_Material [file wxx090_suppl_supplementary_material.pdf]

Online Supplementary Material

Solvent Transfer – Efficiency of Risk Management Measures

Katharina Bluemlein[1], Manfred Elend[1], Tim Meijster[2], Alison Margary[3], Rosalie Tibaldi[4], Stefan Hahn[1], Susanne Hesse[1]

[1] Fraunhofer Institute for Toxicology and Experimental Medicine, Hannover, Germany
[2] Shell Health, Shell International B.V. The Hague , The Netherlands
[3] Shell Health, Shell International Ltd , London , UK
[4] ExxonMobil Biomedical Sciences, Inc., Annandale, NJ , USA

Literature Research – Additional Information

The literature search was conducted using the databases SciFinder®, Scopus and WoS with the combinations of keywords listed in Table S 1, resulting in 1319 hits in SciFinder®, 761 hits in Scopus and 153 hits in WOS (including doublings). All abstracts and titles were screened in combination with the ECEL entries. In addition, a total of 65 publications were considered as complete work. After further refinement and removal of references with missing efficiency and references with no relation to solvents and/or the relevant industry area 28 references remained, including Journals such as Annals of Occupational Hygiene, Indian Journal of Occupational and Environmental Medicine, Journal of Exposure Analysis and Environmental Epidemiology, and Applied Occupational and Environmental Hygiene.

Publications on the following topics were considered to be out of scope for this project and therefore not further evaluated:

- Toxicology based publications (immune effects, drug approval, etc.)
- Efficacy of insecticides
- Exposure to UV radiation or nuclear radiation in power plants,
- “Cost containment”; minimisation of costs
- Containment of diseases (Ebola, SARS etc.)
- Non-occupational exposure (e.g. lead in house dust, environmental exposure)
- Noise exposure
- If RMMs have been investigated in the following ways:
    - quantitative, but no exposure measured (e.g. velocities)
    - only visual examination (e.g. smoke extraction)
    - gradient measurements (e.g. in dependence of distance to hood) – interesting but difficult to use for derivation of efficiency as the personal component is missing. Obviously at a certain distance the “efficiency” will become 100%.
- Influence of exposure to chemical on photosynthesis or other processes, diseases etc.
- Odour containment
- Patents or product descriptions (e.g. new type of drum pump for sale)
- Solvent emission by plants and fungi
- Methane emission by cattle

- Other topics which are obviously not exposure related (e.g. containment of goats with barbed wire, the discipline of space in a Japanese fitness club)

Table S 1 Literature databases and entered keyword combinations

**Keyword combinations SciFinder®**	**Keyword combinations Scopus**	**Keyword combinations WoS**
Exposure reduction drain occupational	Containment efficacy exposure	Containment efficacy exposure
Containment and exposure reduction	Containment efficiency exposure	Containment efficiency exposure
Containment efficacy exposure	Containment emission reduction	Containment emission reduction
Containment efficiency exposure	Control measure solvent	Control measure solvent
Containment emission reduction	Drum pump	Enclosure efficacy exposure
Control measure solvent exposure reduction	Enclosure efficacy exposure	Enclosure efficiency exposure
Control measure solvent	Enclosure efficiency exposure	Enclosure emission reduction
Enclosure efficacy exposure	Enclosure emission reduction	Enclosure exposure reduction
Enclosure efficiency exposure	Enclosure exposure reduction	Exposure reduction maintenance
Enclosure emission reduction	Exposure reduction maintenance	Exposure reduction solvent
Enclosure exposure reduction	Exposure reduction solvent	
Exposure reduction drain		
Exposure reduction flush		
Exposure reduction maintenance occupational exposure		
Exposure reduction solvent occupational		

Table S 2 Equipment used in the laboratory based simulations

Equipment	Supplier
Reservoir container (120 L) with removable lid; two openings: 1) id = 34 cm , 2) id = 4 cm	Ökolube; PN: SP21008
Reservoir container blue (120L) with two bung holes 1.) id = 4cm; 2.) id = 7cm*	FassWulf; PN: K206
Collection container (60 L) with removable lid; two openings: 1) id = 34 cm , 2) id = 4 cm	Ökolube; PN: SP21007
Drum pump set "Solvents"	Lutz Pumpen GmbH
Portable IR-Spectrometer	Asynco – Gasmet FTIR Gas Analyser (Model: DX 4015)
Antistatica Kit	Bürkle; PN: 5602-100
Fume hood 2-451-GAND; dimensions: depth (56.5 / 62 cm – 75 cm), width (114.5 cm -120.5 cm), height (max. 214 cm), performance 0 or 1000-1200 m^3 / hour)	Köttermann (Model: 2-451-GAND)
Gas collection tube / buffer volume (1 L)	

* The bung hole with an id of 7cm was used for the drum pump transfer whereas the second bung hole remained closed at all times.

Table S 3 Experimental results and comparison with suggestions originally used in chemical safety assessments.

				emission reduction (%)			
**Evaluated ES #**	**ES # for comparison**	**experimental aspect represented by exposure reduction**	**Phrase(s) represented by exposure reduction:**	**Minimal**	**Optimal**	**Mean**	**efficiency originally suggested by ESIG and TRA (%)**
**Gravity transfer**							
2: Vented open gravity transfer with partial enclosure (inside open fume cupboard, switched on) into a container, room ventilation	1: Baseline – Gravity transfer from an open container into another open container with no exhaust and no room ventilation system in place	Open fume cupboard (switched on), room ventilation	E60 'Minimise exposure by partial enclosure of the operation or equipment and provide extract ventilation at openings'<br>General room ventilation	98	99.3	98.8	E60: 80 (prof) /90 (industrial)
3: Vented open gravity transfer with full enclosure (inside closed fume cupboard, switched on) into a container, room ventilation	1: Baseline – Gravity transfer from an open container into another open container with no exhaust and no room ventilation system in place	Closed fume cupboard (switched on), general room ventilation	E61 Minimise exposure by extracted full enclosure for the operation or equipment<br>General room ventilation	>99	>99	>99	E61: 90 (prof) /95 (industrial)
4: Gravity transfer from an open container into another open container – application of a local exhaust system (LEV, elephant trunk) and no enclosure (outside fume cupboard)[1], with room ventilation	1: Baseline – Gravity transfer from an open container into another open container with no exhaust and no room ventilation system in place	LEV+room ventilation	E54 'Provide extract ventilation to points where emissions occur'; or<br>E66 'Ensure material transfers are under containment or extract ventilation'<br>General room ventilation	96.5	97.9	97.1	general ventilation: 30 / 70 (good / enhanced; standard ECETOC TRA efficiency)<br>LEV: 75-95 (in general); 80-95 (PROC8a/8b/9) (standard ECETOC TRA efficiency)

[1] LEV efficiency probably lower, 14 - 16.5 % room ventilation efficiency included.

				emission reduction (%)			
**Evaluated ES #**	**ES # for comparison**	**experimental aspect represented by exposure reduction**	**Phrase(s) represented by exposure reduction:**	**Minimal**	**Optimal**	**Mean**	**efficiency originally suggested by ESIG and TRA (%)**
**Drum pump transfer**							
5: Drum pump transfer (closed lids) with no exhaust and no room ventilation system in place – accurate use of drum pump (submerged loading),	1: Baseline – Gravity transfer from an open container into another open container with no exhaust and no room ventilation system in place	Drum pump; (closed container lids – standard for solvent drum transfer)	E53 ‘ Use of drum pump’ (E68: Restrict area of openings to equipment)	91.9	95.2	93.5	E53 (E68): 80 (all uses)
6: Vented drum pump transfer (closed lids) with partial enclosure (inside open fume cupboard, switched on) – accurate use of drum pump (submerged loading) , room ventilation	1: Baseline – Gravity transfer from an open container into another open container with no exhaust and no room ventilation system in place	Drum pump; (closed container lids – standard for solvent drum transfer); open fume cupboard (switched on), general room ventilation	E53 ‘ Use of drum pump’ (E68, ‘Restrict area of openings to equipment’) and E66 ‘Ensure material transfers are under containment or extract ventilation.’	99.3	99.7	99.5	E53 (E68): 80 (all uses)
6: Vented drum pump transfer (closed lids) with partial enclosure (inside open fume cupboard) – accurate use of drum pump (submerged loading), room ventilation	5: Drum pump transfer (closed lids) with no exhaust and no room ventilation system in place – accurate use of drum pump (submerged loading)	Open fume cupboard (switched on)	E66 ‘Ensure material transfers are under containment or extract ventilation.’	89.6	93.2	93.1	E60: 80 (prof) /90 (industrial)
7a: Drum pump (closed lids), no enclosure (outside fume	1: Baseline – Gravity transfer from an open	Drum pump, (closed container lids –	E53 ‘ Use of drum pump’ (E68: Restrict area of	96.2	97.8	96.4	E53/ E68: 80 (all uses) general ventilation: 30 /

				emission reduction (%)			
**Evaluated ES #**	**ES # for comparison**	**experimental aspect represented by exposure reduction**	**Phrase(s) represented by exposure reduction:**	**Minimal**	**Optimal**	**Mean**	**efficiency originally suggested by ESIG and TRA (%)**
cupboard), with room ventilation, but no local exhaust system in place – accurate use of drum pump (submerged loading)[1]	container into another open container with no exhaust and no room ventilation system in place	standard for solvent drum transfer), general room ventilation	openings to equipment)<br>General room ventilation				70 (good / enhanced; standard ECETOC TRA efficiency)
7: Drum pump (closed lids) without enclosure (outside closed fume cupboard), with room ventilation and a local exhaust ventilation system (elephant trunk) in place – accurate use of drum pump (submerged loading)[1]	1: Baseline – Gravity transfer from an open container into another open container with no exhaust and no room ventilation system in place	LEV+general room ventilation + drum pump (closed container lids – standard for solvent drum transfer)	E54 'Provide extract ventilation to points where emissions occur'<br>or<br>E66 'Ensure material transfers are under containment or extract ventilation.'<br>and<br>E53 ' Use of drum pump'<br>(E68, 'Restrict area of openings to equipment'<br>General room ventilation)	98.6	99.1	98.9	general ventilation: 30 / 70 (good / enhanced; standard ECETOC TRA efficiency)<br>LEV: 75-95 (in general); 80-95 (PROC8a/8b/9) (standard ECETOC TRA efficiency)
7: Drum pump (closed lids) without enclosure (outside closed fume cupboard,), with room ventilation and a local exhaust ventilation system (elephant trunk) in place – accurate use of drum pump (submerged loading)[1]	5: Drum pump transfer (closed lids) with no exhaust and no room ventilation system in place – accurate use of drum pump (submerged loading)	LEV + general room ventilation	E54 'Provide extract ventilation to points where emissions occur'<br>or<br>E66 'Ensure material transfers are under containment or extract ventilation.'<br>and	79	85.8	82.8	general ventilation: 30 / 70 (good / enhanced; standard ECETOC TRA efficiency)

				emission reduction (%)			
**Evaluated ES #**	**ES # for comparison**	**experimental aspect represented by exposure reduction**	**Phrase(s) represented by exposure reduction:**	**Minimal**	**Optimal**	**Mean**	**efficiency originally suggested by ESIG and TRA (%)**
			General room ventilation				
**Flushing and draining**							
9: Flushed container with no exhaust system and no room ventilation in place	8: Drained container without flushing with no exhaust system and no room ventilation in place	Working on flushed equipment	E55 'Drain down and flush system prior to equipment break-in or maintenance.'	93.2	96.6	95.2	E55: 90 (industrial)
			E65 'Drain down system prior to equipment break-in or maintenance.'<br>or<br>E81: Drain or remove substance from equipment prior to break-in or maintenance.				E65 or E81: 80 (all uses)
